# Be Thankful

**Published:** 1858-01

**Authors:** 


### BE THANKFUL.

James Graham was born with a natural thumb on the left hand, and but one finger, which was four and a half inches long.

The right hand had two fingers only, one of which was five inches long.

He had never walked, but sat cross-legged like a tailor, until the right leg and thigh had grown together, there being no knee-joint, but there were two knee-pans on the left leg.

The right foot was clubbed, and had but three toes, which were webbed together like those of a duck. The left foot was also clubbed, three-toed, and webbed; but it had only one bone, and that was the heel.

Each leg had but one bone, instead of two. His skull was in some places a quarter of an inch thick; in others, almost as thin as paper.

We have often seen him in Broadway, in a little hand-carriage, which he propelled himself by means of a side crank, like the *velocipeds* of children—so well adapted to invigorating the arms, and developing the muscles of the chest.

Mr. Graham supported himself by his expertness in making dials for clocks and watches. He was a cheerful, quiet, and peaceable man; and the skill he exercised in his business readily secured him employment, at which he worked until he became consumptive: and one evening in May last, finding himself too weak to get home, he made his way into a barber's shop in the Bowery, from which he was taken to a suitable apartment, and died in five days after, at the age of forty-four.

"In a quiet little village on the Western Reserve, in Ohio, there lives a man who, physiologically considered, is certainly one of the wonders of the world. His joints are completely ossified, turned to bone, and he is not capable of making the slightest movement, except alternately opening and shutting two fingers of his right hand. His body is as rigid as iron, and it cannot be bent forward or backward without breaking some of his bones. This singular process of ossification has been going on in his system for more than twenty years. He is now about forty-six years old, and has not had the use of his limbs, so that he could walk, since he was nineteen. Ossification commenced first in his ankle joints, gradually extending itself through his system, until he was entirely helpless. Since that time he has been entirely under his mother's care, and she watches over him with an anxiety which only a mother can feel. When about twenty-six years old, he became entirely blind, from some unknown cause, and has remained so ever since. At about thirty, he suffered greatly from the toothache, and finally he had all his teeth extracted. A year or two afterwards, his finger and toe nails all came off, and were supplied by others growing out from his fingers and toes at right angles, and presenting the appearance of horns. What is still more singular with regard to his nails, if the end of the nail is cut off, it will bleed freely. Such is the condition of this remarkable man at the present time. He has been visited by a great number of scientific men from all parts of the world; but all have failed to give any plausible reason of the cause of his transformation from flesh to bone. Singular as it may appear, although his jaw-bone is firmly set in his head, he not only talks freely, but fluently converses with his friends and those who visit him, on all ordinary topics of the day, and he shows himself well informed and of good mind. He is always cheerful, appears contented and happy, and it seems probable that he will live many years to come."

Forty-nine Years in Bed.—"A man, named Sharpe, died recently in England, in his 79th year, having kept his bed voluntarily forty-nine years. At the time of Sharp's death, the window of his room had never been opened for thirty-four years. In this dreary abode did this strange being immure himself. He constantly refused to speak to any one, and if spoken to, never answered, even those who were his constant attendants. His father, by his will, made provision for the temporal wants of his eccentric son, and so secured him a constant attendant. During the whole period of this self-imposed confinement, he never had any serious illness; the only cause of indisposition those about him can remember being a slight loss of appetite for two or three days, caused apparently by indigestion, and this notwithstanding he ate on the average as much as any farm laborer. Though arrived at the age of 79 years, his flesh was firm, fair, and unwrinkled, and with fat, and his weight was estimated at about 240 pounds."

After this simple narration of scattered facts, we need only repeat the admonition at the head of this article to those of our readers whose bodies are undeformed, who have reasonable health, and a very moderate share of the comforts of life: "*Be Thankful.*"

But to the miserable ingrate, who has no physical deformity, who always has good health when proper care is taken of it, who never knew what it was to be without a comfortable home, who has been always well supplied with food and raiment, who has never lost a child, all whose little ones are growing up in comeliness and health, whose religious advantages are of the highest order, whose kindred are kind, whose social relations are agreeable, and yet whose whole existence, is a snarl; who never ceases to growl at the finer house, the richer dress, the more magnificent equipage of the next-door neighbor; whose first waking word of the day is a complaint; who was scarcely ever known to sit down at table without some expressed disapprobation at the quality or mode of preparation of some article of food; who never soothed the sighings of the prisoner, or waited at the bedside of poverty, or kindly eased the burden of the unfortunate: to such a person we say, read the "facts" over again, and go and thank your Maker in sackcloth and ashes, that he has not long ago swept you out of existence.

